# Breaking the chains in plato’s cave: acute care in general practice

**DOI:** 10.1186/s12875-025-02901-2

**Published:** 2025-06-09

**Authors:** Johannes Rieken, Daniel Hötker, Christoph Strumann, Thomas Kötter, Jost Steinhäuser

**Affiliations:** 1 Institute of Family Medicine, University Medical Center Schleswig-Holstein, Campus Lübeck, Ratzeburger Allee 160, Maria-Göppert Straße 9a, 23538 Lübeck, Germany; 2 Bundeswehrkrankenhaus, Hamburg, Germany

**Keywords:** Urgent medical cases, General practitioners, Primary care, Acute care, Emergency care

## Abstract

**Background:**

It is known that Emergency Departments (EDs) handle lower patient loads during periods when general practitioners (GPs) are on service. However, the acute cases managed by GPs are not well described yet.

**Objective:**

This study aims to assess acute medical cases presented at GP’s offices.

**Methods:**

Medical students documented urgent medical cases as defined by patients during a practice internship in GP practices in Northern Germany, from February to July 2024. Levels of urgency were defined as “not-acute” (could have been treated on another day), “acute” (had to be treated the same day) and “emergency” (had to be treated immediately). Additionally, patient’s concerns and expectations were written down and analyzed using qualitative content analyses.

**Results:**

A total of 523 cases were collected from 53 practices. The majority of cases (82%) were treated solely within the practice. Regarding levels of urgency, 275 (53%) were graded acute, 170 (33%) were not-acute and 70 (13%) cases were graded emergencies. Patients’ motivations for urgently visiting their GP are rooted in fears, motivated by relatives, psychological issues and pain. Expectations range from reassurance to diagnostic examinations to the issuing of a sick leave.

**Discussion:**

GPs play a critical role in managing acute medical cases. The holistic approach of GPs including strategies to cope with uncertainty might be useful to be expanded to more specialties of medicine in the acute care sector.

**Conclusion:**

Motivations and expectations of a visit to a GP practice were diverse but comprehensible. An additional way to address the fears and uncertainties that send patients to the doctor could be through telemedicine offerings and educational initiatives, potentially starting as early as school age.

**Supplementary Information:**

The online version contains supplementary material available at 10.1186/s12875-025-02901-2.

## Background

General practitioners (GPs) also treat acutely ill patients. During evenings, weekends, and holidays, when regular GP practices are closed, patients often turn to emergency departments or out-of-hours services, which place additional strain on medical resources [1]. While structured out-of-hours GP services are in place in Germany through regional on-call systems, they are organizationally and logistically distinct from daytime general practice and may not always be perceived as easily accessible by patients [2]. A steady increase in emergency visits adds pressure on healthcare facilities and policymakers [3]. Despite numerous proposed solutions rooted in hospital-based research, most medical cases are managed in primary care settings [4].

Unlike in the hospital sector, where triage systems for emergency rooms are well-researched and established [5], there is limited research on acute care in the outpatient setting ​ [6]. The nature of urgent cases and patient characteristics in out-of-hours care differ significantly from those encountered during regular office hours, suggesting that solutions developed for out-of-hours care cannot be directly applied to in-practice treatment during normal GP hours​ [2].

Consequently, the role of GPs in managing urgent medical cases in routine care is not well described, despite their position as the primary point of contact in outpatient care [7].

This understanding is essential not only for recognizing the scope of care delivered by GPs but also for informing healthcare policy, improving resource allocation, and identifying opportunities to reduce unnecessary hospital visits. It can help tailor interventions to support patients more effectively within the primary care setting. Therefore, this study aimed to explore the significance GPs provide in acute care.

## Methods

Medical students from the University of Lübeck, Germany undertake a mandatory two-week internship in a GP’s practice during their 5th medical year. Of the 53 participating practices, 47 are located in urban areas and 6 in rural settings. Among them, 26 are group practices, 22 are single-handed-GP practices, and five are ambulatory medical centers. As part of this internship, students were tasked with writing minimum five case reports each. A translated version of the case report file can be found in the appendix.

These reports focused on patients who considered themselves as urgent medical cases. Each case report included anonymized age and gender and three questions about the patients’ views on the reason for the encounter (RFE), their concerns, and their expectations. After the encounter, the treating physicians were asked to formulate the consultation result, including diagnosis, further procedures, and an urgency assessment using a predefined definition of three levels of urgency, namely: “not acute” being a medical issue that would not have necessarily been needed to be seen by a physician on that day, “acute” being an issue that needed to be dealt with on the same day and “emergency” being an issue that needed to be dealt with immediately.

The case reports were transcribed into Excel and coded by a GP (JR) using the International Classification of Primary Care Version 2 (ICPC-2) [8]. Statistical analysis was performed using SPSS, including Fisher’s exact test for categorical and ANOVA for numerical variables. A multivariate ordered logistic regression model was used to assess factors influencing the urgency level.

The qualitative analysis of the case reports was conducted using a structured content analysis based on Mayring’s approach. Initially, the case reports were analyzed deductively using predefined categories derived from the research question and theoretical framework. Additionally, an inductive approach was applied to identify new, previously unconsidered topics. The categories were reviewed and refined in an iterative process to ensure high validity and reliability of the findings. To enhance intersubjective traceability, sample coding was performed by two independent raters (JR, GP and JS, GP). Each case was assigned a pseudonym.

### Patients and public involvement

Patients or the public were not involved in the design, conduct, or dissemination of this study. The study was based on anonymized case reports collected by medical students during routine teaching internships and did not involve direct recruitment or data collection from patients themselves.

## Results

A total of 96 students conducted their internships in 53 different GP practices, 94 students (response rate 98%) provided 523 case reports. Cases of patients who graded their medical issue as not acute (10 cases) were excluded from further analysis of the data reducing the total number of cases to 513.

The qualitative analysis of the case reports revealed a structured categorization of patients’ reasons for visiting their GP. The two overarching categories were: Motivation for the visit and expectations regarding the visit. Patients’ motivations reflected a range of concerns, primarily related to fears, external influences, and psychological factors:

A common motivation for visiting the GP was the fear of serious illness, combined with the expectation that these fears could be addressed through diagnostic measures.

*“Clear focus on fear of myocardial infarction. Past medical history includes coronary artery disease with a previous infarction*,* DES stent placement*,* and hyperlipidemia. History of resuscitation in 2005 and known anxiety disorder. Assessment by the doctor*,* hopefully ruling out a more serious cause of the chest pain.”* (pae8ccfa637).

Psychological issues frequently emerged as a significant motivation for patients seeking medical care. These concerns were often deeply intertwined with professional, financial, and familial stressors, as well as emotional and physical exhaustion.

*“The patient reported various concerns regarding his financial*,* family*,* and professional situation. His greatest worry was his current work situation*,* as shift work was taking a significant physical and emotional toll on him. Additionally*,* interpersonal conflicts with his ex-partner and financial troubles further added to his burden. The patient wished for relief from his sleep problems. He hoped to learn which behavioral measures or medications could help with his complaints. He also requested a certificate confirming his unfitness for shift work.“*(0c184eef53).

Therapy-related fears and expectations for medical reassurance and timely intervention were frequently documented, particularly in cases involving physical injuries or complications.

*“Since the patient could not properly examine the wound herself*,* she was afraid that it might already be infected or not healing well. She wanted a doctor to assess the wound and determine whether the healing process was progressing correctly or if intervention was necessary.”* (2dcb9752b8).

Patients with long-term conditions often presented with multifaceted needs, including pain management and minimizing further medical interventions.

*“Additional medication with [Name of an opioid] for persistent back pain lasting years*,* recently exacerbated*,* accompanied by constipation after starting the treatment*,* with a prior tendency due to wheelchair-bound immobility. […] Hospitalization should urgently be avoided; symptoms need relief*,* and an alternative preparation for constipation should be considered.”* (51222d851b).

Further details are compiled in Table 1.

**Table 1:** Patients’ motivations and expectations

Motivations		Expectations	Apt quotation
**Motivation for the visit to the GP**	Fear of	Worsening of Symptoms	*“That the back pain will get worse and won’t go away.”*
		Serious Illnesses: Myocardial infarction Cancer Stroke	*“The patient fears having a heart attack.”*
		Chronic Conditions	*“Complaints are not improving. Fear that it will become chronic.”*
		Letting Colleagues Down	*“That I will be on sick leave for too long and might even need antibiotics (works in caregiving*,* ‘they need me desperately’).”*
		Hospital Admission	*“Years ago*,* a thrombosis was diagnosed in the right leg*,* fear of having a thrombosis again. Also*,* concern about being admitted to the hospital and having to cancel the rehabilitation stay.”*
	External motivation	Relatives	*“At the urging of his wife*,* who is concerned about the heart symptoms*,* the patient comes for an examination.”*
		Protecting Others	*“She is afraid of infecting her children with the virus. Otherwise*,* she can handle everything.”*
	Psychological	Suicidal thoughts	*“Fear of possessing medication. Would take everything due to severe suicidal thoughts.”*
		Lonelyness	*“Needs support*,* someone to listen to her.”*
	Pain		*“The persistent and recurring headaches that do not improve with [name of a NSAR].”*
**Expectations of the visit to the GP**	Diagnostics	Evaluation	*“Quick clarification of the causes and therapy*,* wants to travel to Italy next week.”*
		Reassurance	*“The patient wanted to have the chest pain checked.”*
		Relief	*“The patient wishes to be told whether everything is fine.”*
		Examinations: Laboratory Tests Ultrasound ECG MRI	*“Blood test for clarification.”*
		Classification	*“Clarification of whether the symptoms indicate allergic bronchitis or Long Covid.”*
	Therapy:	Wound care	*“That a doctor examines the wound to ensure proper healing or to intervene if necessary.”*
		Medication	*“To get a cream for the rash as well as something for stomach cramps and diarrhea.”*
		Psychological	*“Fear of separation and hope for psychotherapeutic support.”*
		Physical	*“Expectation that the doctor will restore mobility through therapy.”*
		Sick leave	*“The patient requests a sick note for a few days.”*
		Pain relief	*“Therapy for pain relief*,* as the pain is no longer bearable.”*
		Remedies (e.g., orthotics, physiotherapy, occupational therapy)	*“Wants a prescription for physical therapy to treat the complaints.”*

Table 2 summarizes details of the characteristics of the study population.

**Table 2:** Sample characteristics, clinical pathways and assessment responsibilities

	Demographic characteristics	*n* (%)
**Gender**	female	291 (56.7%)
	male	209 (40.7%)
	missing	12 (2.3%)
	diverse	1 (0.2)
**Age group**	0–14 years	11 (2.1%)
	15–24 years	55 (10.7%)
	25–64 years	295 (57.5%)
	65 years and over	136 (26.5%)
	Missing Mean	16 (3.1%) 50 years
**Urgency according to physicians**	acute	271 (52.8%)
	not acute	165 (32.2%)
	emergency	69 (13.5%)
	missing	8 (1.6%)
**Clinical pathway**	treated in practice	422 (82.3%)
	referred to a specialist	30 (5.8%)
	admitted to the hospital	44 (8.6%)
	missing	17 (3.3%)
**Number of ICPCs of case**	1	305 (59.5%)
	2	134 (26.1%)
	3	68 (13.2%)
	4	6 (1.1%)
**Responsibility for the assessment of urgency before encounter**	Medical assistant + physician	202 (39.4%)
	Medical assistant + physician + patient	170 (33.1%)
	Medical assistant	87 (17%)
	Medical assistant + patient	31 (6.0%)
	Patient	5 (1.0%)
	Physician	4 (0.8%)
	Physician + patient	2 (0.4%)
	Missing	12 (2.3%)

*N* = 513 patients

The data in Table 2 show that the majority of patients were treated in the practice, with smaller proportions referred to specialists or admitted to a hospital. Over half of the cases were categorized as acute. Most cases were coded with a single ICPC code. Medical assistants were primarily responsible for assessing the urgency of cases, either alone or in combination with physicians and patients.

The results presented in Table 3 provide an overview of the clinical pathways in relation to age, gender, and number ICPC-2 diagnosis codes, divided into three categories: treated in practice, referred to a specialist, and admitted to the hospital. The distribution of these clinical outcomes shows distinct patterns based on the variables.

**Table 3:** Clinical pathway in relation to age, gender and number of ICPC2 codes, N (%)

		Clinical pathway			
		treated solely in practice	referred to specialist	admitted to the hospital	significance
**Age group**	0–14 years	11 (100.0)	0 (0.0)	0 (0.0)	< 0.001
	15–24 years	46 (86.8)	5 (9.4)	2 (3.8)	
	25–64 years	257 (89.2)	18 (6.3)	13 (4.5)	
	65 years and over	97 (75.2)	6 (4.7)	26 (20.2)	
**Gender**	diverse	1 (100.0)	0 (0.0)	0 (0.0)	0.669
	male	175 (87.5)	10 (5.0)	15 (7.5)	
	female	238 (84.1)	19 (6.7)	26 (9.2)	
**Number of ICPC2-codes**	1	253 (84.6)	23 (7.7)	23 (7.7)	0.472
	2	112 (84.2)	6 (4.5)	15 (11.3)	
	3	51 (87.9)	1 (1.7)	6 (10.3)	
	4	6 (100.0)	0 (0.0)	0 (0.0)	
**Patients’ worries**	Fear of hospitalisation	5 (50.0%)	0 (0.0)	5 (50.0%)	0.002

Age was significantly associated with the clinical pathway. Children (0–14 years) were exclusively treated in practice, while older adults (65+) had a significantly higher hospital admission rate (20.2%) compared to younger groups (*p* < 0.001).

The results of the logistic regression model to analyze the factors influencing urgency in general practice cases are shown in Table 4.

**Table 4:** Ordered logistic regression model

Variables	(1)	(2)	(3)
**Age groups** (reference: 65 years and over)	Odds Ratio (*p*-value)	Odds Ratio (*p*-value)	Odds Ratio (*p*-value)
0–14 years	0.17 (0.007)	0.15 (0.005)	0.11 (0.004)
15–24 years	0.33 (< 0.001)	0.3 (< 0.001)	0.26 (< 0.001)
25–64 years	0.71 (0.106)	0.74 (0.169)	0.69 (0.116)
female	0.93 (0.709)	0.88 (0.495)	0.84 (0.393)
**ICPC2-Diagnosis Chapters**			
General and unspecified (A)		1.0 (0.989)	1.09 (0.743)
Digestive (D)		1.86 (0.035)	2.41 (0.006)
Eye (F)		4.44 (0.003)	7.25 (< 0.001)
Ear (H)		0.99 (0.986)	0.91 (0.882)
Cardiovascular (K)		2.23 (0.057)	2.6 (0.035)
Musculoskeletal (L)		1.26 (0.431)	1.31 (0.383)
Neurological (N)		2.01 (0.018)	2.29 (0.01)
Psychological (P)		0.39 (0.085)	0.31 (0.057)
Respiratory (R)		1.44 (0.172)	1.83 (0.039)
Skin (S)		1.45 (0.281)	1.76 (0.132)
Endocrine and metabolic (T)		1.66 (0.662)	1.94 (0.6)
Urological (U)		3.35 (0.016)	4.93 (0.003)
Pregnancy and family planning (W)		-	-
Female genital (X)		0.95 (0.967)	3.82 (0.293)
Male genital (Y)		-	-
Social problems (Z)		0.55 (0.541)	1.26 (0.819)
**GP Practices fixed effects**	no	no	yes
**observations**	473	473	473
**McFadden**	0.019	0.055	0.125
**LOGLIKE**	63.918	449.470	761.633

Younger age groups showed significantly lower odds of being graded more urgent compared to those aged 65 and over. Specifically, for ages 0–14, the odds ratios decreased across models, with the final model showing an odds ratio of 0.11 (*p* = 0.004), indicating an 89% reduction in the odds. Similarly, for ages 15–24, the odds ratio is 0.26 (*p* < 0.001), suggesting a 74% reduction in odds. The 25–64 age group showed no significant effect across models.

Several ICPC2 diagnosis chapters were significantly associated with increased odds of a higher level of urgency. Eye (F) showed the strongest increase with an odds ratio of 7.25 (*p* < 0.001), followed by Urological (U) with an odds ratio of 4.93 (*p* = 0.003). Cardiovascular (K) presents an odds ratio of 2.6 (*p* = 0.035), while Digestive (D) had an odds ratio of 2.41 (*p* = 0.006), and Neurological (N) showed an odds ratio of 2.29 (*p* = 0.01). Respiratory (R) became significant in the final model with an odds ratio of 1.83 (*p* = 0.039). Psychological (P) approached significance but remained marginal, suggesting a potential reduction in odds without strong evidence.

## Discussion

The present study provides a first insight into acute cases managed in routine care in GP practices, focusing on patients’ motivations as their RFE and evaluating the urgency levels assigned by physicians.

Being trained both in inpatient as well as in outpatient care, GPs are skilled in acute medicine, managing a wide array of urgent cases that often parallel those requiring hospital admission. The broad spectrum of patients is inherent to the discipline, as GPs treat individuals of all ages, from infants to the elderly [7]. The collected case reports document a comprehensive range of medical conditions, illustrating the diverse health issues that GPs must address. In contrast, most other medical specialists in the ambulatory sector typically handle a smaller scope of different diseases in their daily work [9]. Notably, the vast majority of acute cases were managed exclusively within the practices. This mirrors findings in chronic care, where GPs similarly handle the majority of cases independently [10]. The diversity of urgency levels represents a distinctive aspect of the work environment that sets GPs apart from other medical professionals in the ambulatory setting [11, 12].

The data collected on responsibility for the assessment of urgency before encounter show that medical assistants (MFAs) play an important role. They are often the first point of contact for patients, performing initial assessments, supporting physicians in clinical tasks, and handling administrative duties [13, 14]. Their role is essential in ensuring efficient patient flow and supporting the high patient volumes typical in general practice.

A relevant finding from our data is the considerable discrepancy between patients’ and physicians’ perceptions of urgency. This gap reflects a common phenomenon in acute medicine, where patients’ anxiety, concerns, and subjective views may differ from the clinical judgment of healthcare providers. Studies have focused on this discrepancy in the emergency department (ED) and out-of-hours care settings [6, 15, 16].

An institution designed for treating urgent medical cases, such as an ED, is well equipped for high-grade, urgent situations. However, it is not intended to manage patients with objectively low-grade medical issues coupled with a high level of anxiety and perceived urgency [17]. A discrepancy which often results in frustration on both sides: on the part of the medical staff, who feel their professional and timely capacities are invested suboptimal, and on the part of the patients, who do not always feel taken seriously [18].

One approach to help guiding these patients can currently be found in telemedicine care, which gives patients access to GPs via a secure connection on their end devices. This system reduces the strain on in-person facilities, such as ED, and also helps patients who perceive their condition as urgent to receive timely reassurance and guidance remotely [19].

A strategy to reduce the number of patients with objectively non-urgent medical conditions binding traditional ED resources is the integration of GPs in hospital work [20]. A GP specialist brings several additional competencies to healthcare, some of which impact critical outcomes such as mortality, costs, and overall patient outcomes [21]. Therefore, given the current GP shortage in many Western countries, it is questionable whether they should be employed in hospitals where only part of their broad expertise is used [22].

To find a different solution to the problem of ED overcrowding might be expedient to look at the skillset GPs use to deal with these cases competently. As symptoms in primary care are often more diffuse than in patients with high-grade medical emergencies, finding a definite diagnosis is often not adequate [23]. This dictates the need to endure diagnostic uncertainty, a key skill in general practice. Hospitals are equipped with almost any measure of diagnostic possibility and hospital staff are used to using it. Diagnostic uncertainty cannot thrive in an environment designed to rule out any form of doubt by default. There is growing evidence that diagnostic tests in hospital settings are frequently used on patients who could have been safely managed in general practice [24]. Studies have shown that tests such as chest X-rays or blood panels are often conducted in emergency departments to exclude serious conditions, even when clinical examination and vital signs may suffice [25]. This practice reflects a low tolerance for diagnostic uncertainty in hospital environments and can contribute to unnecessary healthcare costs and patient anxiety [26]. By contrast, GPs frequently manage similar cases without these investigations, relying on their longitudinal knowledge of the patient and their ability to conduct risk assessments based on clinical judgment [23].

A comprehensive understanding of the patient’s RFE is fundamental to an effective treatment [7]. The specialization of secondary care has resulted in a reduction in the holistic and general approach to patient care [27, 28]. Resulting in miscommunication, this narrow scope can complicate treatment ultimately - even putting patients at risk. Physicians must identify and address the original RFE of their patients, ensuring these aspects are integrated into the diagnostic and therapeutic process for truly effective care [29]. However, a rotation in general medicine plays virtually no role in the training of emergency physicians yet [30–32].

Teaching GP skills to non-GP physicians might be a feasible approach to deal with the shortcomings in the handling of lower-grade medical emergencies in acute care instead of implementing GPs into hospital care [33]. Including a mandatory rotation into general medicine for any type of physician treating walk-in patients at the ED might not immediately reduce patient loads in the amount the implementation of experienced GPs did [20], but might be a strategy to reintroduce a generalist approach.

An additional way to address the fears and uncertainties that send patients to the doctor might be through educational initiatives, potentially starting as early as school age. Teaching fundamental knowledge about the functioning of the human body, the development and prognosis of diseases, warning signs, and the structure of the healthcare system - including the appropriate points of contact [17] - could have a relieving effect on the healthcare system in the future.

### Strengths and Limitations

The chosen methods were suitable to deeply explore motives and expectations of patients seeking acute care. The documented urgency levels allow for an appreciation of the highly individual assessment that is necessary for each encounter.

The progressive approach to model-building in this study is a key strength, allowing for a clear understanding of how different factors - age, gender, ICPC-2 codes, and GP practices - independently and collectively influence urgency outcomes. This method also highlights the complexity of urgent care management in general practice, where multiple factors intersect to shape clinical decisions.

However, there are limitations to consider. The reliance on case reports written by medical students introduces potential variability in data collection, as the students may have differing levels of experience and interpret patient interactions in diverse ways. The qualitative assessment of the patient’s worries and expectations was not recorded but relies entirely on the documentation of the medical students. Another limitation is that patient classification was restricted to symptom level, as a final diagnosis could not be documented in every case. the patient cases included were selected by medical students during their internships, potentially introducing selection bias. The relatively low number of pediatric cases observed in this study may reflect the urban setting of most participating practices, where it is common for children to be treated by specialized pediatricians rather than general practitioners. In contrast, in rural areas of Germany, GPs frequently manage pediatric patients themselves [34]. Additionally, a possible source of bias could be that medical students may feel more hesitant to document cases involving children compared to adult patients, which could further contribute to the underrepresentation of pediatric cases in the study.

The total patient population of the participating practices was not documented, which limits the generalizability of the findings. Additionally, the quantitative results presented here should be viewed as exploratory, given the non-randomized selection of cases and the absence of a broader patient population baseline. Future research should aim to include a more representative sample and standardized methods for case selection to confirm these findings as well as include patient follow-ups to comprehensively assess the entire clinical pathway.

## Conclusion

Identifying motivations and expectations within a patient contact is crucial to meet patients’ demand. Enduring diagnostic uncertainty might be something GPs could teach other specialties involved in acute care. Patients should get offered additional services e.g., by telemedicine to relieve the pressure on emergency departments. Finally, teaching on school level, when to address which sector of care, might be promising.

## Electronic Supplementary Material

Below is the link to the electronic supplementary material.

**Supplementary Material 1**: **Appendix**

## Data Availability

The datasets generated and/or analyzed during the current study are not publicly available but are available from the corresponding author upon reasonable request.

## Declarations

### Patient and public involvement

Patients and/or the public were not involved in the design, conduct, reporting, or dissemination plans of this research.

## Abbreviations

ED: Emergency department
GP: General practitioner
ICPC: International classification of primary care
RFE: Reason for encounter
