# Supplementary figures and images for: Breaking the chains in plato’s cave: acute care in general practice
Source: BMC Prim Care. 2025 Jun 9;26:198. doi: 10.1186/s12875-025-02901-2 (PMC12147272; doi:10.1186/s12875-025-02901-2)

## Appendix


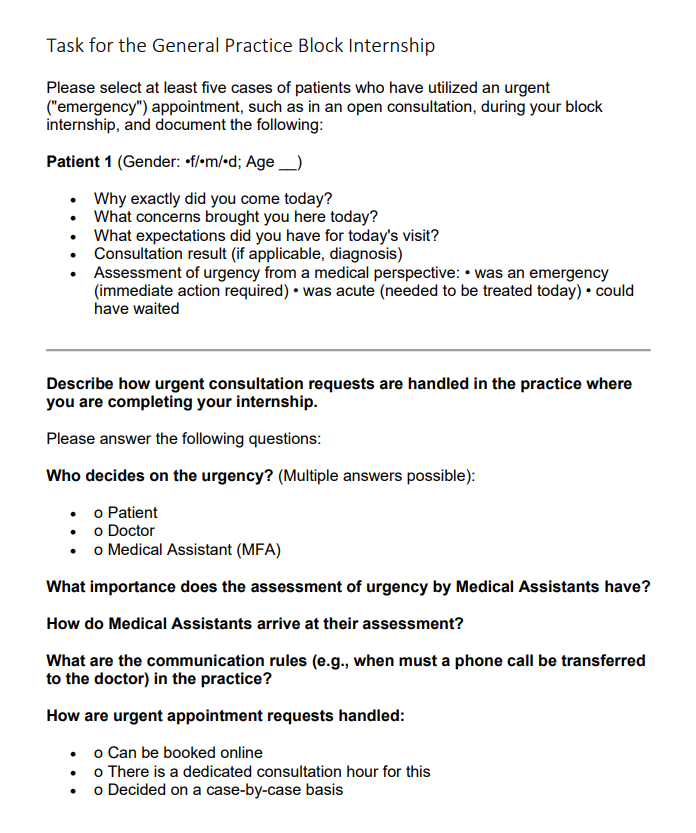

Supplement: Supplementary file 1 — Supplementary Material 1: Appendix [file 12875_2025_2901_MOESM1_ESM.docx]
